# Using the Computer-based Health Evaluation System (CHES) to Support Self-management of Symptoms and Functional Health: Evaluation of Hematological Patient Use of a Web-Based Patient Portal

**DOI:** 10.2196/26022

**Published:** 2021-06-08

**Authors:** Jens Lehmann, Petra Buhl, Johannes M Giesinger, Lisa M Wintner, Monika Sztankay, Lucia Neppl, Wolfgang Willenbacher, Roman Weger, Walpurga Weyrer, Gerhard Rumpold, Bernhard Holzner

**Affiliations:** 1 University Hospital of Psychiatry II Medical University of Innsbruck Innsbruck Austria; 2 Oncotyrol – Center for Personalized Cancer Medicine Innsbruck Austria; 3 University Hospital of Psychiatry I Medical University of Innsbruck Innsbruck Austria; 4 Internal Medicine V: Haematology & Oncology Innsbruck University Hospital Innsbruck Austria; 5 Comprehensive Cancer Center Innsbruck Innsbruck Austria; 6 University Clinic of Medical Psychology Medical University of Innsbruck Innsbruck Austria; 7 Evaluation Software Development GmbH Innsbruck Austria

**Keywords:** quality of life, monitoring, patient portals, multiple myeloma, chronic lymphocytic leukemia, patient-reported outcome measures, eHealth, mHealth

## Abstract

**Background:**

Patient portals offer the possibility to assess patient-reported outcome measures (PROMs) remotely, and first evidence has demonstrated their potential benefits.

**Objective:**

In this study, we evaluated patient use of a web-based patient portal that provides patient information and allows online completion of PROMs. A particular focus was on patient motivation for (not) using the portal. The portal was developed to supplement routine monitoring at the Department of Internal Medicine V in Innsbruck.

**Methods:**

We included patients with multiple myeloma and chronic lymphocytic leukemia who were already participating in routine monitoring at the hospital for use of the patient portal. Patients were introduced to the portal and asked to complete questionnaires prior to their next hospital visits. We used system access logs and 3 consecutive semistructured interviews to analyze patient use and evaluation of the portal.

**Results:**

Between July 2017 and August 2020, we approached 122 patients for participation in the study, of whom 83.6% (102/122) consented to use the patient portal. Patients were on average 60 (SD 10.4) years old. Of patients providing data at all study time points, 37% (26/71) consistently used the portal prior to their hospital visits. The main reason for not completing PROMs was forgetting to do so in between visits (25/84, 29%). During an average session, patients viewed 5.3 different pages and spent 9.4 minutes logged on to the portal. Feedback from interviews was largely positive with no patients reporting difficulties navigating the survey and 50% of patients valuing the self-management tools provided in the portal. Regarding the portal content, patients were interested in reviewing their own results and reported high satisfaction with the dynamic self-management advice, also reflected in the high number of clicks on those pages.

**Conclusions:**

Patient portals can contribute to patient empowerment by offering sought-after information and self-management advice. In our study, the majority of our patients were open to using the portal. The low number of technical complaints and average time spent in the portal demonstrate the feasibility of our patient portal. While initial interest was high, long-term use was considerably lower and identified as the main area for improvement. In a next step, we will improve several aspects of the patient portal (eg, including a reminder to visit the portal before the next appointment and closer PROM symptom monitoring via an onconurse).

## Introduction

Patient-reported outcomes (PRO) are defined as all reports about the health status given directly by the patient without interpretation of the patient’s response by a clinician or anyone else [1]. While they have long been used in clinical trials, they have, in recent decades, also progressed to enriching routine clinical care [2,3]. Driven by technological progress and an increased availability and use of the internet in the population [4], it has become easier to incorporate the patient’s perspective into clinical care using electronic patient-reported outcome measures (ePROMs).

In oncological care, PROs can support patient-clinician communication [5,6] and aid early detection of symptoms [7,8] and have been linked to a decrease in hospitalization and emergency department visits [9]. Building on the evidence base showing the benefits of PRO use at the hospital, the use of web-based solutions to assess ePROMs outside the hospital has gained traction. In the last decade, the number of web-based patient portals that enable the completion of ePROMs has risen and recent research has demonstrated the potential benefits of patient portals in large-scale clinical trials [10-13]. For example, in a randomized controlled trial by Denis et al [10], web-based symptom monitoring was associated with increased survival compared to standard imaging surveillance following treatment for lung cancer. The authors argue that web-based symptom monitoring may allow for earlier symptom detection and appropriate reaction by health care professionals (HCPs). Moreover, PRO web monitoring can be highly cost-effective [11] and reduce the administrative burden of assessments inside the hospital, as patients can complete ePROMs from home. Finally, remote assessments are especially helpful in an outpatient setting, as assessments conducted on the day of chemotherapy administration at the hospital have shown to systematically underestimate patients’ symptom burden associated with treatment [14].

Despite the benefits shown in study settings, and even though detailed guidance on how to implement PROs into clinical practice exists [15,16], electronic PRO monitoring and especially patient portals are still only occasionally adopted in routine clinical practice. This can be attributed to the limited integration into electronic health records, a lack of financial reimbursement for ePROM assessments, and a lack of standardized assessment methods, which hinder implementation in routine care [2]. Patient portals also vary considerably regarding the focus of the implementation and their goals and use of PROs [17]. More research is needed that evaluates the usability and acceptability of different applications in routine practice to extend and strengthen the evidence base in this heterogeneous and evolving field of research.

At the Department of Internal Medicine V in Innsbruck, a patient portal for outpatients with multiple myeloma (MM) and chronic lymphocytic leukemia (CLL) was developed to conduct remote PRO assessments. The primary aims for development of the portal were to enhance patient empowerment, encourage patient engagement with PROs, and reduce the administrative burden of PRO assessments inside the hospital.

In this study, we evaluated patient use of the various components of the portal and aimed to identify patient lack of motivation for not using the patient portal and potential barriers to accessibility.

## Methods

### Study Design

In our observational, longitudinal study, we evaluated patient use of the portal based on two data sources: semistructured interviews conducted during 3 consecutive visits to our unit after introducing eligible patients to the patient portal and system access logs recording the duration of user sessions and how often each page of the web portal was accessed.

### Description of the PRO Monitoring and the Patient Portal

In June 2016, we implemented routine ePROM assessments to supplement care of outpatients with MM at the Department of Internal Medicine V in Innsbruck. The two primary aims of the implementation of ePROM assessments were to supplement the Austrian Myeloma Registry (AMR) with PRO data and enrich clinical care with the data [18]. In July 2017, patients with CLL were added as a second patient group. The use of the Computer-based Health Evaluation System (CHES) [19] enables immediate processing and graphical representation of the results. For monitoring at the hospital, patients complete PRO measures before their medical appointment, and the results are presented to the HCP prior to the consultation. The implementation and feasibility of the assessments at the hospital and use of data from patients have been evaluated in the past for the AMR [18], and more details on HCP use of the system are presented elsewhere [20]. This study builds upon our previous implementation strategy and is focused on process evaluation, refinement, and extension (as described in phase IV of the implementation process described by Sztankay et al [18]).

**Figure 1:**
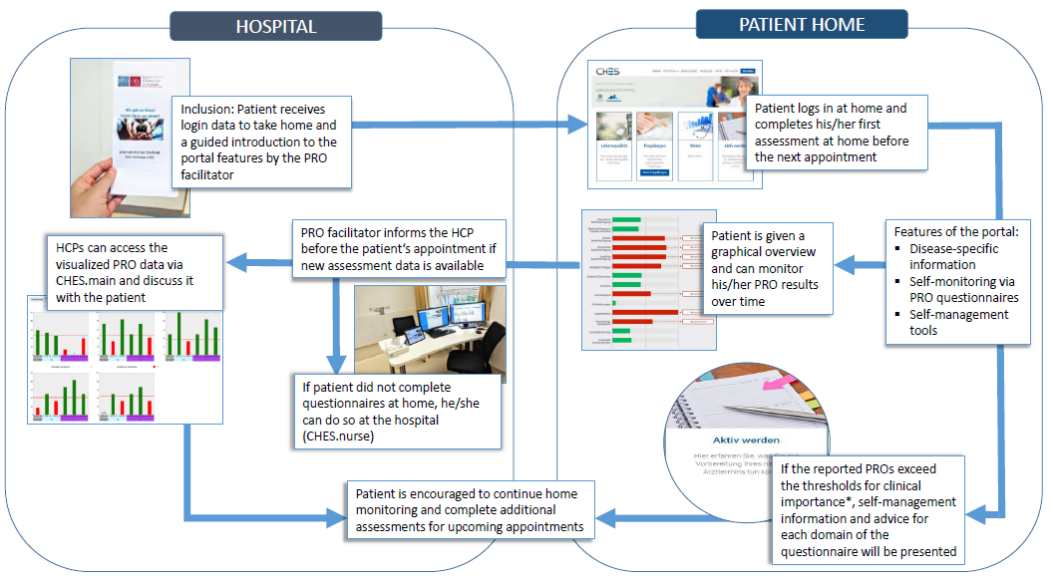
Computer-Based Health Evaluation System patient portal functions when patients are at home and in the hospital. *Thresholds for clinical importance [21] are used to highlight domains that require discussion with the health care provider. PRO: patient-reported outcome; HCP: healthcare professional.

There are 3 main components of CHES: (1) the HCP interface (CHES.main), which presents PRO and patient data to HCPs; (2) the survey interface (CHES.nurse), where the patients complete questionnaires at the hospital; and (3) the patient portal (CHES.portal), which allows remote questionnaire completion and access to supplemental information and self-management advice. See Figure 1 for a visualization of the patient portal and its functionality. See Multimedia Appendix 1 for screenshots of the software and portal.

In this paper, we assess patient use of the patient portal, which features the following functionalities:

- Disease-specific information on CLL and MM: diagnosis, possible symptoms, possible treatments, and links to further information and self-help groups
- PRO assessments with the cancer-specific European Organisation for Research and Treatment of Cancer Quality of Life Questionnaire (EORTC QLQ-C30) in combination with the disease-specific modules EORTC QLQ-MY20 for patients with MM and EORTC QLQ-CLL17 for patients with CLL. The EORTC QLQ-C30 is the most frequently used cancer-specific questionnaire [22] and can be used to measure patient symptoms, functional health, and global quality of life. The modules QLQ-MY20 and QLQ-CLL17 supplement the QLQ-C30 and cover disease-specific issues of quality of life for MM (eg, future perspective, treatment side effects) and CLL patients (eg, symptom burden due to disease and/or treatment, worries/fears regarding health and functioning).
- PRO score review by patient: results are displayed as colored bar charts (longitudinal and cross-sectional). Results that exceed the thresholds for clinical importance [21] are colored red, and results that do not exceed these threshold are colored green (see Lehmann et al [20] for more information).
- Self-management tools and tailored information: based on EORTC QLQ-C30 data, patients are presented with self-management tools for the symptoms and functional health domains. If a patient reports a potentially clinically important result [21], they are directed to the self-management tools (see Lehmann et al [20] for more information). Patients are reminded that in case of severe impairments, they should contact the clinical team directly.

At the outpatient clinic, patients are introduced to the portal by a PRO facilitator (ie, person responsible for the assessments at the hospital). The role of the PRO facilitator is that of a study assistant with a background in psychology or nursing who is trained in the use of PRO data and motivates both patients and HCPs to use the PRO data. Patients are given instructions for use and their personal log-in data for accessing the portal at home by the PRO facilitator.

Typically, patients are advised to complete ePROM assessments in the week before their hospital visits. Visits are scheduled at regular intervals that range between 1 week and 12 months, depending on the disease stage and treatment plan. Patients are encouraged to complete questionnaires as often as they like, even on a daily basis. Questionnaires completed within 7 days of a hospital visit are used to inform the HCP of the patients’ health status and linked to the clinical data in the AMR. Data from assessments in between hospital visits are used for research purposes and enable more continuous tracking of patients’ health status in the AMR.

Although patients participating in the routine monitoring are encouraged to use the patient portal to report PROs, they can still complete assessments at the hospital (eg, if they forgot to use the portal before their hospital visit).

### Study Sample

We defined the following inclusion criteria for participation in monitoring via the patient portal: fluency in German, age 18 years or older, diagnosis of MM or CLL, current treatment at the outpatient clinic of the Department of Internal Medicine V or the Comprehensive Cancer Center Innsbruck of the Medical University of Innsbruck, consent given to routine PRO monitoring at the hospital, and completion of at least one ePROM during a prior visit to the hospital.

Patients were deemed ineligible if they had no access to a computer or the internet or lacked sufficient knowledge to log into a website using a username and password. All patients provided informed consent to use the patient portal. Patients who declined to use the portal were asked for the reason for their refusal. The study and use of patient data are covered by the ethics approval for the AMR issued by the ethics committee of the Medical University of Innsbruck (study number AN3252 266/4.2 386/5.14).

At baseline, demographics (sex, age, marital status, education level, and employment status) and experience with the internet and computer technology (frequency, duration) were collected via a questionnaire. Diagnosis and cancer stage were obtained from the hospital’s medical records.

### Selection of Outcome Measures

We used different sources to evaluate patient use and perception of the patient portal. Table 1 displays the selection of outcome measures.

**Table 1:** Selection of outcome measures.

Questions addressed	Outcome measure	Assessment method	Data type
How often do patients use the portal? Is the portal feasible for use during routine clinical care?	ePROM^a^ completion rate (number of completed ePROMs before study time points)	Assessed via CHES^b^	Quantitative data
Why do patients use (or not use) the portal? What is their feedback on portal components?	Patient perspectives on portal components and motivation to use (or not use) the portal; accessibility barriers identified	Semistructured interviews	Qualitative and quantitative data/questions
How often do patients log into the portal? How long do patients log into the portal in a single session? Which pages are viewed and how often?	Patient user patterns in the portal: number and duration of log-ins per patient; portal page views	Assessed via CHES portal log data	Quantitative data

^a^ePROM: electronic patient-reported outcome measure.

^b^CHES: Computer-based Health Evaluation System.

### Patient Interviews

**Figure 2:**
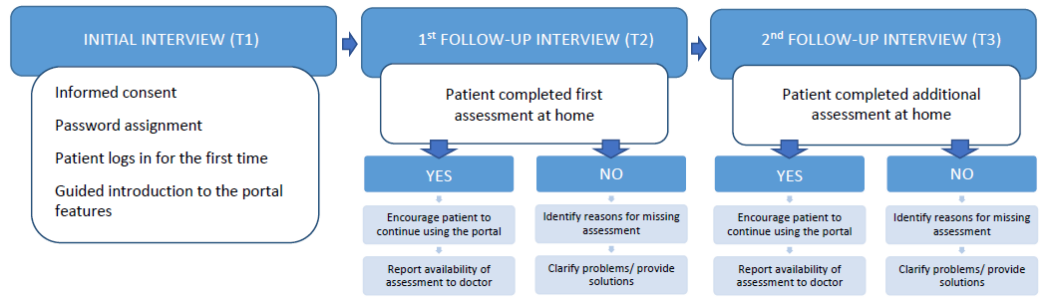
Patient interview procedure and topics.

Each patient participated in 3 semistructured face-to-face interviews conducted by authors LN, PB, and JL following a fixed schedule during 3 consecutive visits to the outpatient unit. See Figure 2 for a flowchart of the interview process. The topics covered in the interviews were as follows:

T1: Guided introduction to the portal and user log-in

- Possible difficulties logging in
- Navigating the portal
- Reviewing PRO results
- Other suggestions/remarks by patients

T2: Evaluation of use: acceptability and usability

- Satisfaction with instructions for completion of questionnaire
- Navigating the portal
- Technical difficulties
- Reviewing PRO results and accessing self-help tools
- Relevance and usefulness of provided content

T3: Evaluation of use: acceptability and usability

- Technical difficulties
- Interest in content not explored during previous log-ins
- Satisfaction with design
- Other suggestions/remarks by patients

### ePROM Completion Rate

To evaluate initial interest in the portal, we assessed the proportion of patients in routine monitoring at the hospital consenting to use the patient portal at T1. To evaluate continued use, we assessed the proportion of patients who completed ePROMs prior to hospital visits at T2 and T3; ePROMs were considered to be linked to a hospital visit if they were completed in the 7 days before the visit.

### CHES Log Data

The frequency (absolute number) and duration (in minutes) of the use of the website were determined by the CHES system log. These data were gathered irrespective of the interview time points each time the patient logged into the system. Log data were collected per session but were not linked to individual patients. Therefore, patients with more sessions have a greater weight in this analysis. Log-ins that occurred on the same day were considered a single session, and durations of these sessions were summed.

### Data Analysis

Sociodemographic and clinical data were analyzed at time point T1. Comparison of patients completing ePROMs via the patient portal and of those who declined to use the portal were made with *t* tests (for parametric variables) and chi-square tests (for nonparametric variables).

The interviewer took field notes during the interviews and for each open question. Responses from the interviews were either analyzed descriptively (for yes/no questions) or paraphrased and category-coded (for open answers) independently by two researchers (JL and PB) and harmonized by discussion in case of different coding. We translated selected quotes into English for the results section of this paper.

We also analyzed the frequency (number) of views for each portal page, excluding views on the start page, where patients are directed automatically after logging in. Further, we calculated the time patients needed to complete the questionnaires online (mean completion time per patient averaged across all patients).

## Results

### Participant Enrollment and Baseline Characteristics

**Figure 3:**
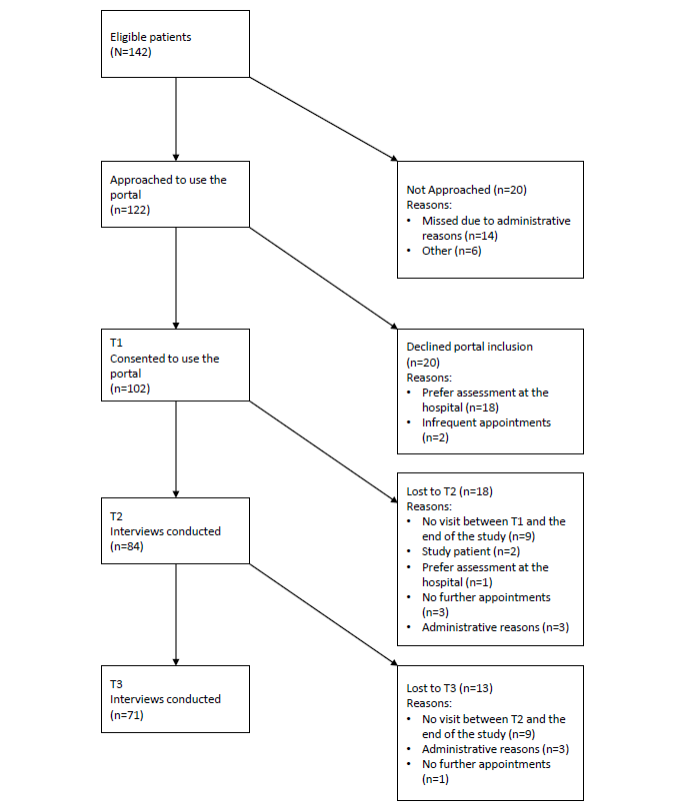
Recruitment flowchart (study patient denotes patients who were also participating in other clinical studies and were not included in our study so as not to overburden the patient with clinical questionnaires).

Recruitment began in July 2017 and was open until August 2020. During the study period, we identified 142 eligible patients already participating in the electronic PRO monitoring in the hospital, of whom 85.9% (122/142) were approached for study participation and use of the patient portal. Of those, 83.6% (102/122) consented to be included in the patient portal. Of 20 patients not willing to use the patient portal, 18 patients stated a preference of questionnaire completion only at the hospital as the reason. The complete enrollment process is shown in Figure 3.

The full sociodemographic information and clinical data are given in Table 2. There were no statistically significant differences regarding age, sex, education, time since the initial diagnosis, and type of internet use between those who agreed and those who refused to participate (all *P*>.09). The age range of patients included in the portal was 39 to 83 years, and the age range of patients not included in the portal was 39 to 77 years. A statistically significant difference was found for general internet use (see Table 2), with participants who consented to use the portal reporting higher internet use than those who did not consent to use the portal: of patients who used the portal, 94% (94/100) reported using the internet at least multiple times per week compared to 76% (17/22) for patients who did not use the portal.

**Table 2:** Sociodemographic information.

Characteristic			Included in patient portal (n=102)		Not included in patient portal (n=23)		Statistic			*P* value
							χ^2^	*t* score		
**Sex, n (%)**			—^a^		—		0.1	—		.80
	Female	37 (37)		9 (39)		—		—	—	
	Male	65 (64)		14 (61)		—		—	—	
Age (years), mean (SD)			59.9 (10.5)		63.2 (10.5)		—	1.33		.19
**Diagnosis, n (%)**			—		—		4.0	—		.047
	Multiple myeloma	63 (62)		9 (39)		—		—	—	
	Chronic lymphocytic leukemia	39 (38)		14 (61)		—		—	—	
Time since diagnosis (years), mean (IQR)			4.5 (0.9-6.8)		4.7 (0.9-6.6)		—	1.73		.09
**Highest education, n (%)**			—		—		0.9	—		.84
	Compulsory or lower	9 (9)		1 (5)		—		—	—	
	Vocational training	55 (55)		10 (50)		—		—	—	
	High school certificate	18 (18)		4 (20)		—		—	—	
	University	18 (18)		5 (25)		—		—	—	
	Missing data^b^	2		3		—		—	—	
**Internet use (type), n (%)**			—		—		0.7	—		.40
	Private use only	66 (65)		17 (74)		—		—	—	
	Job and private use	36 (35)		6 (26)		—		—	—	
**Internet use (frequency), n (%)**			—		—		10.0	—		.02
	>Once per month	2 (2)		0 (0)		—		—	—	
	Multiple times a month	4 (4)		5 (23)		—		—	—	
	Multiple times a week	30 (30)		4 (17)		—		—	—	
	Daily	64 (64)		13 (59)		—		—	—	
	Missing data^b^	2		1		—		—	—	

^a^Not applicable.

^b^Missing values were not included in the calculation of percentages.

### Interviews

Interviews lasted between 10 and 30 minutes. Only 5% (5/102) patients required help from the PRO facilitator because they were not able to read their username and password (small font size). After the log-in and during the first interview, no patients reported technical difficulties or had difficulties navigating the portal or the questionnaire. Table 3 shows the number of completed questionnaires before the interviews and the reasons for noncompletion. Of the patients who participated in all 3 interviews, 37% (26/71) completed the questionnaires prior to the T2 and T3 interview, while the others completed the questionnaires only at the hospital visit.

Table 4 shows the use and evaluation of the portal as reported by patients who completed questionnaires prior to the interviews. Patients who completed questionnaires prior to their follow-up appointments reported using the portal in different ways: the percentage of patients reading additional portal content (eg, disease-specific information) declined over time from 71% (27/38) at T2 to 41% (13/32) at T3. The self-management tools were used and deemed useful by 50% (17/34) of patients at the second and 42% (13/31) at the third interview.

**Table 3:** Questionnaire completion rate and reasons for not completing questionnaires in the patient portal before hospital visits.

Completion rate or reason for noncompletion			Interview 1 (n=102), n (%)		Interview 2 (n=84), n (%)		Interview 3 (n=71), n (%)
**Questionnaire completed before appointment**							
	Yes	102 (100)		38 (45)		32 (45)	
	No	—^a^		46 (55)		39 (55)	
**Reasons not completed**							
	Forgot	—		25 (29)		18 (25)	
	Technical difficulties	—		9 (11)		0 (0)	
	Loss of log-in data	—		0 (0)		3 (4)	
	Study patient^b^	—		0 (0)		1 (1)	
	Lack of time/motivation	—		5 (6)		6 (9)	
	Preferred assessment at hospital	—		3 (4)		7 (10)	
	Other	—		4 (5)		4 (6)	

^a^Not applicable.

^b^Study patients were also participating in other clinical studies using the same questionnaires or similar; they sometimes confused questionnaires from other studies with our study’s questionnaires.

**Table 4:** Use and evaluation of the patient portal as reported in the interviews.

Patient-reported behavior and evaluation of the portal			Completed questionnaires, n (%)				
			Interview 1 (n=102)		Interview 2 (n=38)		Interview 3 (n=32)
**Reading (additional) portal content**							
	No	—^a^		27 (71)		13 (41)	
	Yes	—		11 (29)		18 (56)	
**Looking at one’s own results**							
	Yes	96 (96)		25 (69)		—	
	No	4 (4)		11 (31)		—	
	Missing data^b^	2		2		—	
**Self-management tools**							
	Inspected self-management tools and found them to be useful	—		17 (50)		13 (42)	
	Inspected self-management tools and did not find them useful	—		4 (12)		2 (6)	
	Did not inspect self-management tools	—		13 (38)		16 (52)	
	Missing data^b^	—		4		1	
**Reason for not inspecting self-management tools**							
	No impairments reported^c^	—		6 (18)		8 (26)	
	Lack of time	—		1 (3)		1 (3)	
	Other	—		6 (18)		7 (23)	

^a^Not applicable.

^b^Missing values were not included in the calculation of percentages.

^c^If a patient did not report impairments above the thresholds for clinical importance, the software did not suggest viewing self-management tools when looking at their own results.

### Patient Comments

Patients were encouraged to provide additional comments following their answers to the interview questions. In the first interview, patients reported high satisfaction with the presentation of the results as bar charts.

...bar charts are a good way of presenting the results. I like that I can compare my results to those of other patients with cancer.

I like that I can see my results after having completed the questionnaire.

Patients who did not inspect their own results expressed that they were feeling fine and therefore had no interest in viewing their results.

Similarly, patients who were feeling fine or reported no impairments did not inspect the self-management tools, while others provided positive feedback on the self-management tools or the color-coding of questionnaire results.

I did not check the self-management advice because I am not feeling ill. Why would I check it?

I like the design. The arrow in the results [direct link from the results to the self-management information] was very helpful.

...liked the color coding [red/green] as it was simple and easy to understand.

Four patients expressed the wish for a reminder (email or text message) before the next appointment to complete the questionnaires online. Two patients mentioned that they would like to be able to choose their password or username themselves (which the software currently does not allow). Three patients also explicitly reported a decline in motivation toward the end of the study, caused by a lack of sufficient feedback from physicians who did not discuss their PRO results during the consultations.

### CHES Log Data

Over the study period, we registered a total of 796 sessions (ie, log-ins by patients). Of the patients who logged into the patient portal, 27% (28/102) logged in once, 18% (18/102) logged in twice, 9% (9/102) logged in 3 times, 15% (16/102) logged in 4 to 6 times, 14% (14/102) logged in 7 to 10 times, and 17% (17/102) logged in more than 10 times (total range of 1-57).

The mean duration of a session was 9.4 (median 6, range 1-90) minutes. It took patients on average 2.9 minutes to complete the EORTC QLQ-C30, 1.8 minutes to complete the EORTC QLQ-MY20, and 1.5 minutes to complete the EORTC QLQ-CLL17. This adds up to an average questionnaire completion time of 4.7 minutes for patients with MM and 4.4 minutes for patients with CLL.

**Figure 4:**
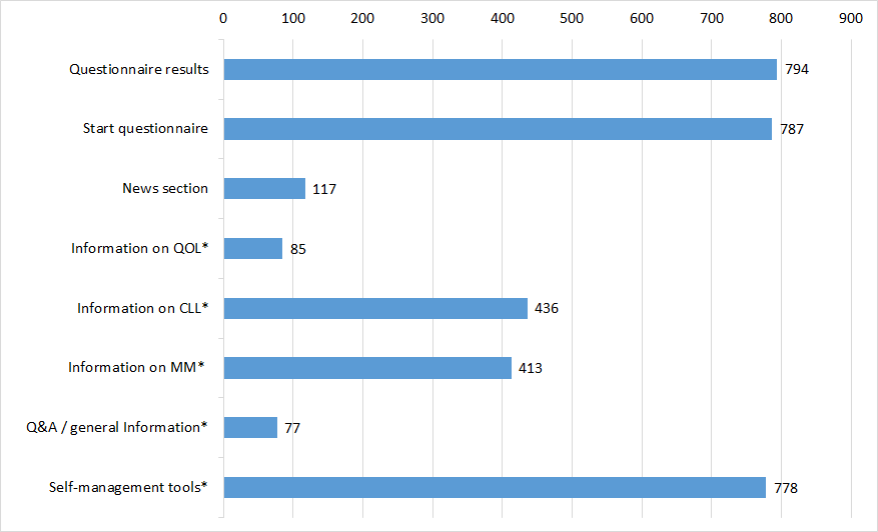
Page views in the patient portal by category (excluding home page). Categories marked with an asterisk combine multiple pages and subpages. QOL: quality of life; MM: multiple myeloma; CLL: chronic lymphocytic leukemia; Q&A: questions and answers.

During an average session, patients viewed 5.3 (median 4, range 1-28) different pages. Figure 4 shows how often specific components of the patient portal were viewed. A total of 3487 views were registered. The most frequently viewed self-management pages were those providing information on dyspnea (89 views), diarrhea (80 views), cognitive functioning (64 views), and emotional functioning (49 views). The least frequently viewed self-management pages were those providing information on obstipation (11 views), role functioning (12 views), social functioning (21 views), and pain (18 views). Multimedia Appendix 2 shows the view count on all self-management pages.

## Discussion

### Principal Findings

In our study, we evaluated the use of the patient portal in patients with MM and CLL participating in routine electronic PRO monitoring. We found that in those patients who were eligible to use the patient portal, the majority (84%) were willing to use the portal. Only a few users reported difficulties logging in, but no users reported problems navigating the survey. However, we found that considerably fewer patients (37%) than initially included adopted a continued use of the portal across all 3 study time points. On average, patients spent 9.4 minutes in the portal per session.

### Uptake of Home Monitoring

Our recruitment rate (ie, patients consenting to try the patient portal) is high compared to rates found in other studies. For example, other feasibility or usability studies report that between 21% and 64% [23-25] of patients were willing to use patient portals. Our high recruitment rate is most likely a consequence of the fact that patients were already participating in electronic PRO monitoring and so had already approved some form of monitoring. We would like to note the high inclusion rate for PRO monitoring at the hospital, which was, as reported previously, 94% of all MM patients treated in the department [18]. Our study emphasizes the possible synergy between assessments inside the hospital and home monitoring; patients who had already participated in electronic PRO monitoring inside the hospital were open to also using a patient portal. Moreover, the high acceptance of the patient portal might have been induced by the opportunity to get accustomed to ePROMs at the hospital with the help of a PRO facilitator before being introduced to the patient portal. We hypothesize that such a stepwise approach can reduce potential reservations about using electronic measures and contribute to user empowerment.

The adoption of home monitoring by a considerable proportion of patients also meant that those patients did not need to be assessed at the hospital. In this way, the patient portal reduced the administrative burden of assessing those patients’ PROs at the hospital and allowed us to allocate those resources elsewhere.

### Long-Term Use of the Portal

Over the course of the study, slightly less than half of the participants used the portal prior to their follow-up appointments. The main reason reported by patients was that they had forgotten to report their PRO data using the patient portal. Especially for patients with an infrequent appointment schedule (intervals between hospital visits of up to 12 months), forgetting to use the portal is, in fact, not surprising. This issue might be addressed by implementing an automated reminder (email or text message) as has been done with similar home monitoring systems [24,26]. For example, the AmbuFlex system in Denmark has, in the past, used different forms of reminders, including letters, emails, and text messages. They ultimately implemented communication via a national secure email program, which accounts for 93.2% of communication to patients and secures high completion rates [27].

Additionally, it is important to note that due to our routine monitoring approach, ePRO data from patients who did not use the portal prior to the follow-up appointments were not lost. Instead, those patients were invited to complete the ePROMs at the hospital as was done with other patients who did not use the portal.

While only a few of our patients directly mentioned a decline in motivation to complete ePROMs due to HCPs not picking up on the results during the consultations, this is in fact a frequent problem of PRO implementations in clinical settings [3,26,28,29]. For patients, there is little perceived benefit of completing the questionnaires if the results are not reviewed and discussed by HCPs. Instead, sharing their health status via questionnaires might even become burdensome. Therefore, it is important to engage HCPs with the concept of PROs and train them in the use of PRO results to prevent PRO data from becoming meaningless busywork that hinders clinical practice instead of enhancing it [28]. This requires the education and training of HCPs, which can be achieved by conducting specialized training programs [30]. A buy-in strategy may be used to increase HCP engagement with PROs, for example, via the adoption of the user interface to HCP needs and preferences, regular meetings, or analyses of PRO data in the registry upon request of HCPs. Another important approach is to make PRO data comprehensible and actionable for HCPs (eg, by providing advice on how to react to results) [26,31,32]. In our system, this is done, for example, by using thresholds for clinical importance for the EORTC QLQ-C30 [21] that facilitate the interpretation of patient PRO data.

Finally, the literature shows that in order to provide a sound theoretical basis for sustainable PRO solutions in routine care settings, an implementation science approach can be followed [33]. Implementation science can help identify barriers (which are often similar across contexts) and enablers (which often depend on the given hospitals’ context) of patient-reported outcome measure (PROM) implementations.

### User Patterns in the Portal

As has been found in other studies [13,17,25], the majority of our patients found the display of self-management advice linked with their results to be valuable. A recent review of electronic systems to measure PROs found that less than one-third (29%) of published electronic systems include features that provide tailored automated advice to patients, and less than half (41%) provide general educational information [17]. In a qualitative study evaluating another eHealth application, tailored feedback and advice was rated as appealing by most participants [34]. Participants valued the option of accessing information remotely between appointments and having a low threshold to receiving such information (compared to having to consult their treating physician or having to search for information) [34]. These are notions that echo the high number of views of pages with self-management advice we found in our study.

Nevertheless, despite the approval for self-management advice we found in our study, a recent clinical trial showed that, while patient portals can contribute to improved health-related quality of life [13], their measurable benefits on patient activation (knowledge, skills, and confidence for self-management) could not be shown in a diverse sample of cancer survivors [13]. However, previous evidence suggested that such effects may be more pronounced in newly diagnosed patients [25,35]. Another randomized controlled trial showed that weekly PRO monitoring along with the provision of tailored self-management advice, compared to usual care, significantly enhanced self-efficacy in patients with cancer [36].

### Limitations

Our study was designed to be carried out during routine care with minimal interruption of the clinical workflow. Therefore, interview time points were integrated into the patients’ hospital visits. This resulted in varying intervals between interviews (between 1 week and 1 year in a few cases), which may have influenced the results. However, we point out that these are real-world visitation schedules, and any application designed for routine care should be evaluated accordingly. Similarly, we included more males than females in our study. This reflects the epidemiology of MM and CLL, and the male/female ratio was comparable to data from the AMR.

Second, the CHES log data did not allow for detailed analysis of individual user page-view patterns but only for analysis of overall page views and duration and frequency of sessions. This means that patients logging on to the portal more frequently have a greater weight in the analysis of page views. Moreover, page view numbers have to be interpreted with care as they provide no information on whether the pages were actually read.

Another limitation is that the interviews were conducted by the authors (LN, PB, and JL), who introduced patients to the portal. Therefore, some patients might have been reluctant to voice criticism in the interviews, even though we actively encouraged patients to also report negative feedback.

Finally, we consider some sample-related limitations: We included only German-speaking patients in the patient portal. While the EORTC questionnaires are available in a large number of languages (and can be completed at the hospital [20]), translating and updating the content of the portal would require considerable resources. This results in a potential bias, and our findings may not be generalizable to patients with other first languages and limited German language proficiency. We are aware that this can create an imbalance in the provision of care, as this systematically excludes certain patient groups. In fact, patients not speaking the primary language of the country might profit most from receiving disease-specific information and from being able to report symptoms online in their native language. Moreover, we observed a selection bias due to patients’ age (patients with MM were on average 11 years younger than the mean age of patients in the AMR), as older patients might be less proficient using the internet and were consequently not included in our study. While this is an important limitation to consider, its impact should decrease over time, as the population’s internet capabilities have been steadily increasing [37] and should further increase in the years to come.

### Future Steps

Following the insights gained in this study, we are currently in the process of updating our monitoring procedure and software. One important step is to more closely monitor patients’ PROMs to swiftly identify deteriorations and act accordingly; we aim to have a trained onconurse monitor patient results and check on patients in case of deteriorations. If necessary, the nurse can schedule an earlier appointment or alert the treating HCP. Another planned improvement is to implement an email or text message reminder to improve PROM completion rates.

### Conclusion

Our study shows that a patient portal enabling remote PRO data assessment can complement routine electronic PRO implementation at the hospital by reducing the burden of administration for the clinical team and offering an additional way for patients to engage with PROs. We found that the majority of patients were open to using the patient portal and interested in assessments from home. The low number of technical problems and absence of complaints demonstrate the general user-friendliness of our portal. While initial uptake was high, fewer patients adopted regular use of the portal prior to their appointments. To increase long-term participation rates, further motivational (eg, increasing HCP engagement with the PRO data) and technical (eg, email reminder) measures are needed.

## Appendix

Multimedia Appendix 1 Examples of pages in the patient portal.

Multimedia Appendix 2 Total view count in the portal and specific view count for the self-management pages.

## Abbreviations

AMR: Austrian Myeloma Registry
CHES: Computer-Based health Evaluation System
CLL: chronic lymphocytic leukemia
EORTC QLQ–C30: European Organisation for Research and Treatment of Cancer Quality of Life Questionnaire
ePROM: electronic patient-reported outcome measure
HCP: health care professional
MM: multiple myeloma
PRO: patient-reported outcome
PROM: patient-reported outcome measure
