# Supplementary material for: Using the Computer-based Health Evaluation System (CHES) to Support Self-management of Symptoms and Functional Health: Evaluation of Hematological Patient Use of a Web-Based Patient Portal
Source: J Med Internet Res. 2021 Jun 8;23(6):e26022. doi: 10.2196/26022 (PMC8262597; doi:10.2196/26022)
Supplement: Multimedia Appendix 1 [file jmir_v23i6e26022_app1.pdf]

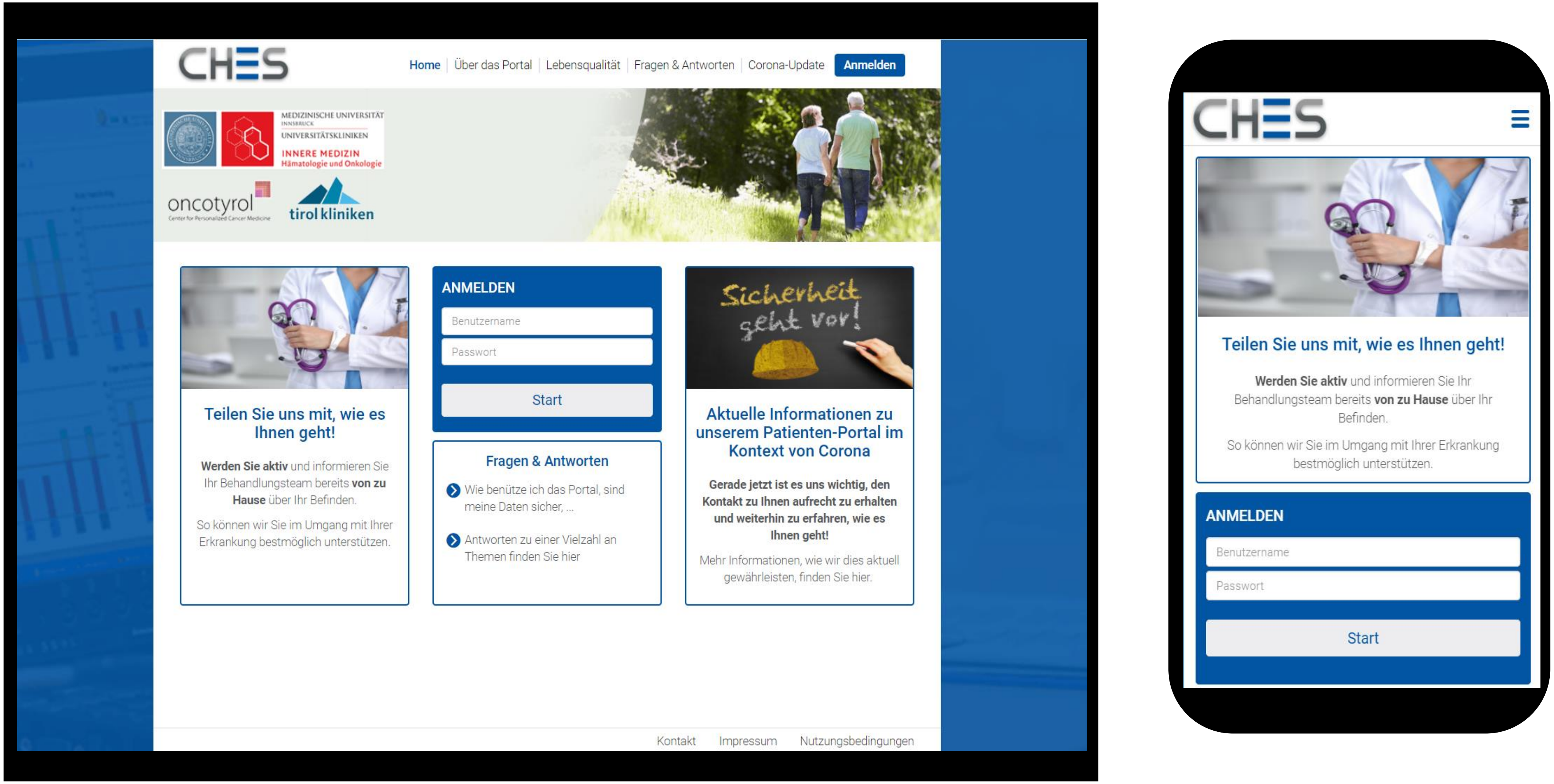

CHES
Home
Über das Portal
Lebensqualität
Fragen & Antworten
Corona-Update
Anmelden
MEDIZINISCHE UNIVERSITÄT INNSBRUCK
UNIVERSITÄTSKLINIKEN
INNERE MEDIZIN
Hämatologie und Onkologie
oncotyrol
tirol kliniken
Teilen Sie uns mit, wie es Ihnen geht!
Werden Sie aktiv und informieren Sie Ihr Behandlungsteam bereits von zu Hause über Ihr Befinden.
So können wir Sie im Umgang mit Ihrer Erkrankung bestmöglich unterstützen.
ANMELDEN
Benutzername
Passwort
Start
Fragen & Antworten
Wie benütze ich das Portal, sind meine Daten sicher, ...
Antworten zu einer Vielzahl an Themen finden Sie hier
Sicherheit geht vor!
Aktuelle Informationen zu unserem Patienten-Portal im Kontext von Corona
Gerade jetzt ist es uns wichtig, den Kontakt zu Ihnen aufrecht zu erhalten und weiterhin zu erfahren, wie es Ihnen geht!
Mehr Informationen, wie wir dies aktuell gewährleisten, finden Sie hier.
Kontakt
Impressum
Nutzungsbedingungen
CHES
Teilen Sie uns mit, wie es Ihnen geht!
Werden Sie aktiv und informieren Sie Ihr Behandlungsteam bereits von zu Hause über Ihr Befinden.
So können wir Sie im Umgang mit Ihrer Erkrankung bestmöglich unterstützen.
ANMELDEN
Benutzername
Passwort
Start

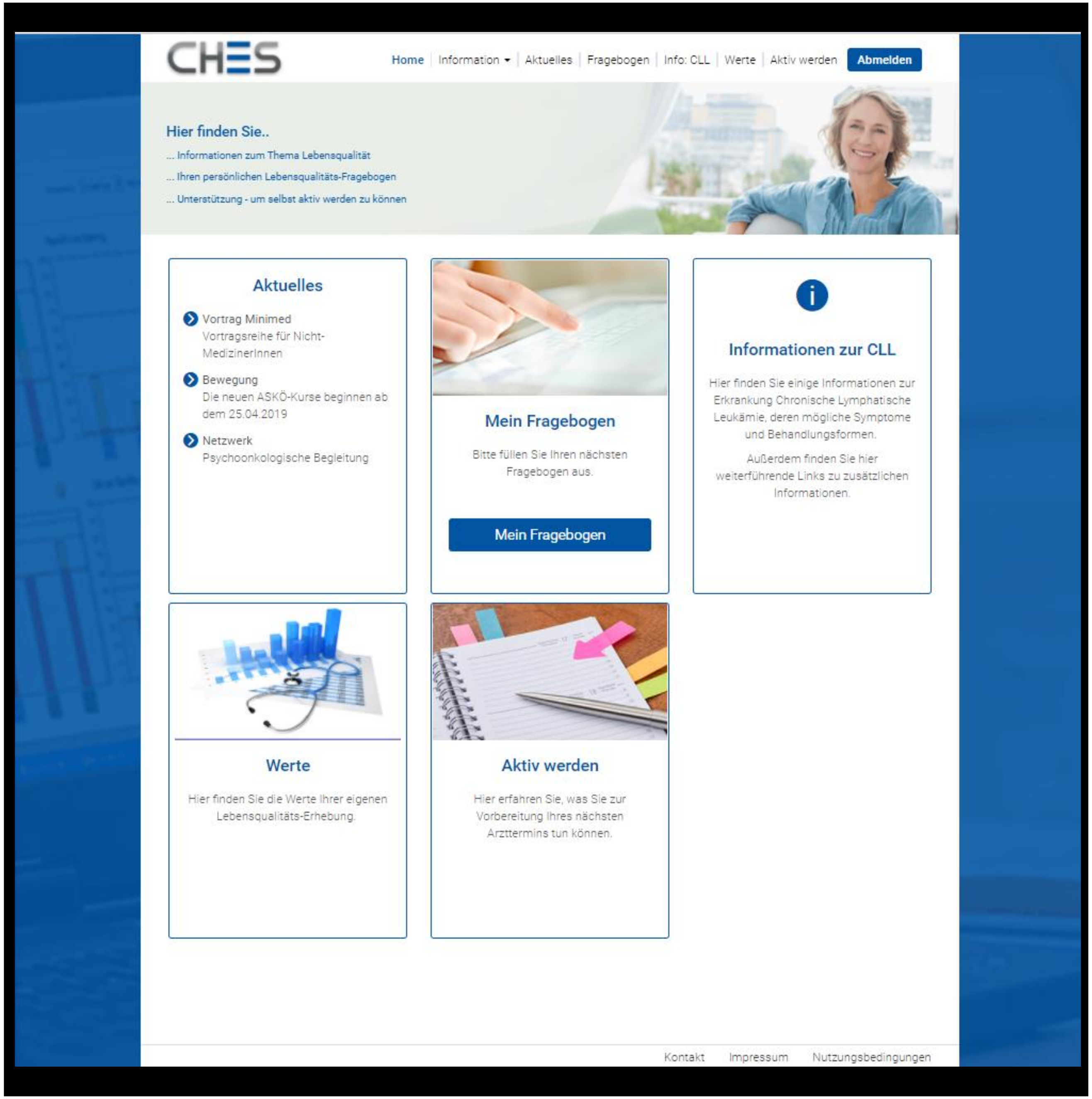
CHES
Home | Information | Aktuelles | Fragebogen | Info: CLL | Werte | Aktiv werden | Abmelden
Hier finden Sie..
... Informationen zum Thema Lebensqualität
... Ihren persönlichen Lebensqualitäts-Fragebogen
... Unterstützung - um selbst aktiv werden zu können
Aktuelles
Vortrag Minimed
Vortragsreihe für Nicht-MedizinerInnen
Bewegung
Die neuen ASKÖ-Kurse beginnen ab dem 25.04.2019
Netzwerk
Psychoonkologische Begleitung
Mein Fragebogen
Bitte füllen Sie Ihren nächsten Fragebogen aus.
Mein Fragebogen
Informationen zur CLL
Hier finden Sie einige Informationen zur Erkrankung Chronische Lymphatische Leukämie, deren mögliche Symptome und Behandlungsformen.
Außerdem finden Sie hier weiterführende Links zu zusätzlichen Informationen.
Werte
Hier finden Sie die Werte Ihrer eigenen Lebensqualitäts-Erhebung.
Aktiv werden
Hier erfahren Sie, was Sie zur Vorbereitung Ihres nächsten Arzttermins tun können.
Kontakt
Impressum
Nutzungsbedingungen

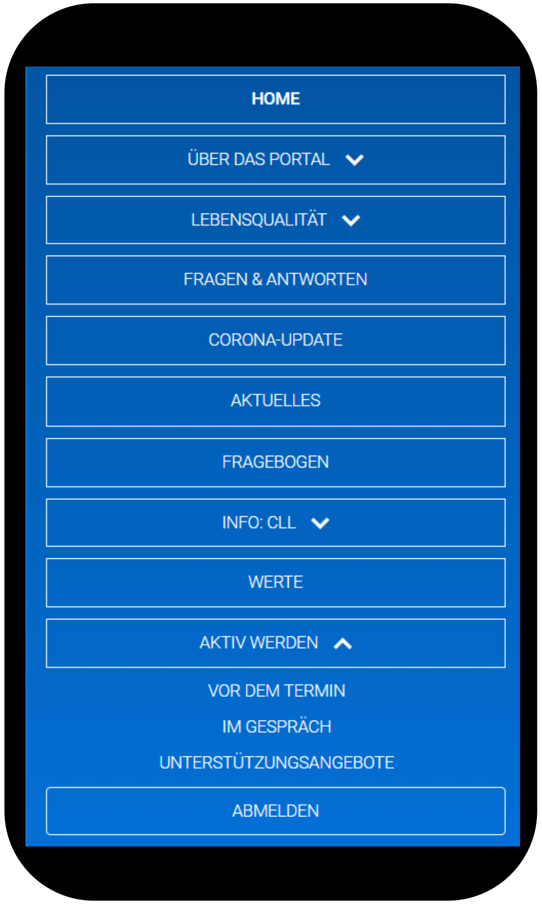
HOME
ÜBER DAS PORTAL
LEBENSQUALITÄT
FRAGEN & ANTWORTEN
CORONA-UPDATE
AKTUELLES
FRAGEBOGEN
INFO: CLL
WERTE
AKTIV WERDEN
VOR DEM TERMIN
IM GESPRÄCH
UNTERSTÜTZUNGSANGEBOTE
ABMELDEN

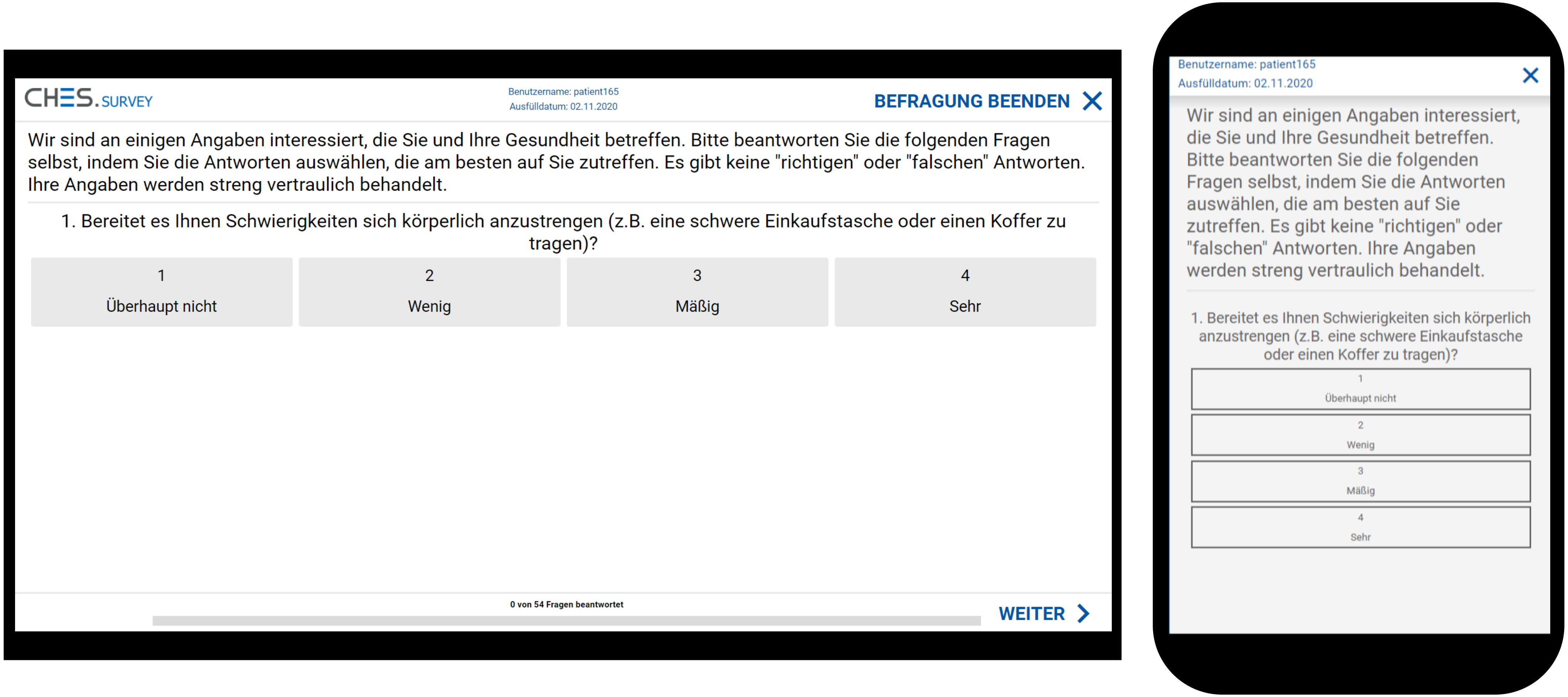
CHES.SURVEY
Benutzername: patient165
Ausfülldatum: 02.11.2020
BEFRAGUNG BEENDEN
Wir sind an einigen Angaben interessiert, die Sie und Ihre Gesundheit betreffen. Bitte beantworten Sie die folgenden Fragen selbst, indem Sie die Antworten auswählen, die am besten auf Sie zutreffen. Es gibt keine "richtigen" oder "falschen" Antworten. Ihre Angaben werden streng vertraulich behandelt.
1. Bereitet es Ihnen Schwierigkeiten sich körperlich anzustrengen (z.B. eine schwere Einkaufstasche oder einen Koffer zu tragen)?
1 Überhaupt nicht
2 Wenig
3 Mäßig
4 Sehr
0 von 54 Fragen beantwortet
WEITER
Benutzername: patient165
Ausfülldatum: 02.11.2020
Wir sind an einigen Angaben interessiert, die Sie und Ihre Gesundheit betreffen. Bitte beantworten Sie die folgenden Fragen selbst, indem Sie die Antworten auswählen, die am besten auf Sie zutreffen. Es gibt keine "richtigen" oder "falschen" Antworten. Ihre Angaben werden streng vertraulich behandelt.
1. Bereitet es Ihnen Schwierigkeiten sich körperlich anzustrengen (z.B. eine schwere Einkaufstasche oder einen Koffer zu tragen)?
1 Überhaupt nicht
2 Wenig
3 Mäßig
4 Sehr

Informationen zur CLL

- Einführung
- Informationen zur Erkrankung
- Diagnose der Chronischen Lymphatischen Leukämie (CLL)
- Symptome der Chronischen Lymphatischen Leukämie (CLL)
- Behandlung der Chronischen Lymphatischen Leukämie
- Nach Abschluss der Behandlung
- Nützliche Links
- Quellen

Was versteht man unter der Erkrankung "Chronische Lymphatische Leukämie"?

Mit dem **Begriff "Chronische Lymphatische Leukämie"** (CLL) wird eine bösartige Erkrankung der weißen Blutkörperchen, genauer der Zellen des lymphatischen Systems bezeichnet. Sie kann das Immunsystem und die Blutbildung im Knochenmark beeinträchtigen.

Wie der Name „*chronisch*" bereits andeutet, handelt es sich bei der CLL um eine Erkrankung mit meist langsamem und schleichendem Verlauf.

Das „*lymphatische*" System ist neben dem Blutkreislauf ein weiteres Transportsystem, das über den ganzen Körper verteilt ist. Es setzt sich aus den Lymphgefäßen, den lymphatischen Organen (Milz, Leber, Thymus und Mandeln) und den Lymphknoten, welche als Filter der Lymphflüssigkeit dienen, zusammen. Die Zellen des lymphatischen Systems (Lymphozyten) werden als Bestandteil der Lymphflüssigkeit (Lymphe) über Lymphbahnen im ganzen Körper transportiert.

„*Leukämie*" ist ein Sammelbegriff für Erkrankungen, die mit einer bösartigen Zunahme weißer Blutkörperchen (Lymphozyten) im Blut einhergehen. Die Lymphozyten bilden Antikörper gegen Erreger von Infektionskrankheiten und sind so ein wichtiger Teil der Immunabwehr, die durch die Erkrankung beeinträchtigt ist.

Bei einer CLL-Erkrankung werden zu viele B-Lymphozyten (bestimmte weiße Blutkörperchen) gebildet, die allerdings funktionell unbrauchbar sind. Während der Entwicklung der Lymphozyten in den Lymphknoten kommt es zu einem Fehler und eine erste entartetet Krebszelle entsteht. Diese Zelle beginnt sich unkontrolliert zu vermehren und gibt die bösartigen Genveränderungen weiter. Die so entstehenden kranken, funktionsuntüchtigen Lymphozyten verdrängen nach und nach die gesunden Zellen im Knochenmark. Dadurch wird die Produktion von funktionstüchtigen Antikörpern behindert und die Immunabwehr geschwächt.

Je nachdem, wo die bösartig veränderten Zellen gefunden werden, spricht man entweder von einer SLL („small lymphocytic lymphoma"; B-Lymphozyten überwiegend im Lymphknoten) oder einer CLL (Lymphomzellen vor allem im Blut).

Die Chronische Lymphatische Leukämie ist die häufigste Form der Leukämie im Erwachsenenalter und betrifft vor allem PatientInnen in fortgeschrittenem Alter. Jedes Jahr erkranken in Europa etwa 3-5 von 100.000 Menschen. Männer haben ein fast doppelt so hohes Erkrankungsrisiko wie Frauen.

Die **Erkrankungshäufigkeit** steigt ab dem Alter von 50 Jahren deutlich an, wobei die meisten Diagnosen ab dem 70. Lebensjahr und durchschnittlich mit 73 Jahren gestellt werden. Erkrankungen vor dem 55. Lebensjahr sind selten, allerdings werden durch die Zunahme routinemäßiger Vorsorgeuntersuchungen immer öfter auch jüngere PatientInnen diagnostiziert (ca. 10%).

In seltenen Fällen (ca. 10%) kann es zu einer Richter-Transformation kommen. Dabei geht die CLL stetig in eine andere, schnell wachsende Form eines Lymphoms über (z.B. DLBCL). Bei Verdacht auf eine derartige Transformation werden weitere Untersuchungen von Blutbild, Lymphknoten und Milz nötig.

Die **Ursachen** für die Entstehung einer CLL sind noch weitgehend unbekannt. Bestimmte Umweltgifte oder gar Lebensgewohnheiten (incl. Nahrung) konnten nicht als krankheitsauslösende oder –begünstigende Faktoren identifiziert werden.

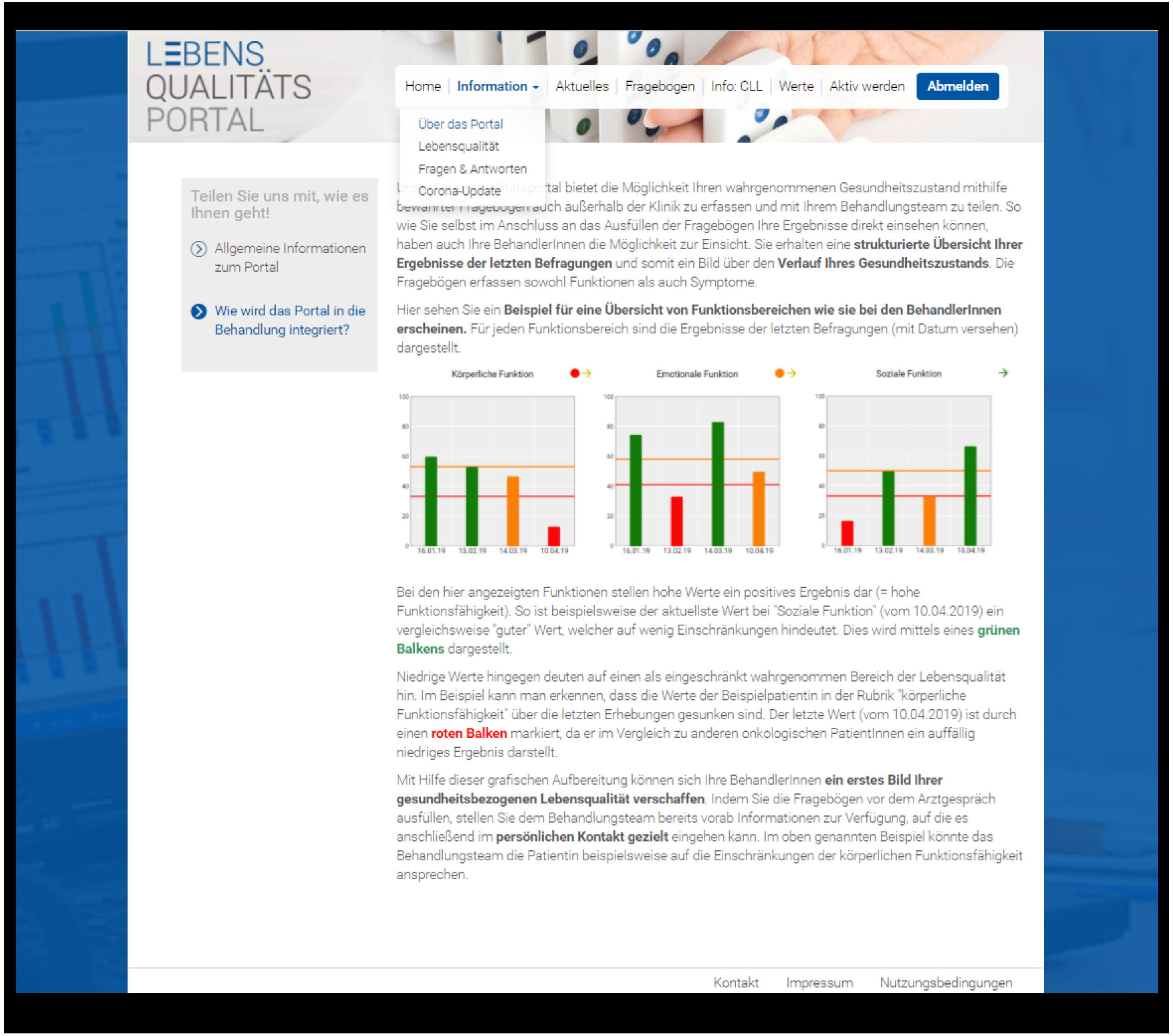

LEBENS QUALITÄTS PORTAL

Home | **Information** | Aktuelles | Fragebogen | Info: CLL | Werte | Aktiv werden | **Abmelden**

- Über das Portal
- Lebensqualität
- Fragen & Antworten
- Corona-Update

Teilen Sie uns mit, wie es Ihnen geht!

- Allgemeine Informationen zum Portal
- Wie wird das Portal in die Behandlung integriert?

U[illegible]tal bietet die Möglichkeit Ihren wahrgenommenen Gesundheitszustand mithilfe [illegible] auch außerhalb der Klinik zu erfassen und mit Ihrem Behandlungsteam zu teilen. So wie Sie selbst im Anschluss an das Ausfüllen der Fragebögen Ihre Ergebnisse direkt einsehen können, haben auch Ihre BehandlerInnen die Möglichkeit zur Einsicht. Sie erhalten eine **strukturierte Übersicht Ihrer Ergebnisse der letzten Befragungen** und somit ein Bild über den **Verlauf Ihres Gesundheitszustands**. Die Fragebögen erfassen sowohl Funktionen als auch Symptome.

Hier sehen Sie ein **Beispiel für eine Übersicht von Funktionsbereichen wie sie bei den BehandlerInnen erscheinen.** Für jeden Funktionsbereich sind die Ergebnisse der letzten Befragungen (mit Datum versehen) dargestellt.

Körperliche Funktion | Emotionale Funktion | Soziale Funktion

Bei den hier angezeigten Funktionen stellen hohe Werte ein positives Ergebnis dar (= hohe Funktionsfähigkeit). So ist beispielsweise der aktuellste Wert bei "Soziale Funktion" (vom 10.04.2019) ein vergleichsweise "guter" Wert, welcher auf wenig Einschränkungen hindeutet. Dies wird mittels eines **grünen Balkens** dargestellt.

Niedrige Werte hingegen deuten auf einen als eingeschränkt wahrgenommen Bereich der Lebensqualität hin. Im Beispiel kann man erkennen, dass die Werte der Beispielpatientin in der Rubrik "körperliche Funktionsfähigkeit" über die letzten Erhebungen gesunken sind. Der letzte Wert (vom 10.04.2019) ist durch einen **roten Balken** markiert, da er im Vergleich zu anderen onkologischen PatientInnen ein auffällig niedriges Ergebnis darstellt.

Mit Hilfe dieser grafischen Aufbereitung können sich Ihre BehandlerInnen **ein erstes Bild Ihrer gesundheitsbezogenen Lebensqualität verschaffen**. Indem Sie die Fragebögen vor dem Arztgespräch ausfüllen, stellen Sie dem Behandlungsteam bereits vorab Informationen zur Verfügung, auf die es anschließend im **persönlichen Kontakt gezielt** eingehen kann. Im oben genannten Beispiel könnte das Behandlungsteam die Patientin beispielsweise auf die Einschränkungen der körperlichen Funktionsfähigkeit ansprechen.

Kontakt Impressum Nutzungsbedingungen

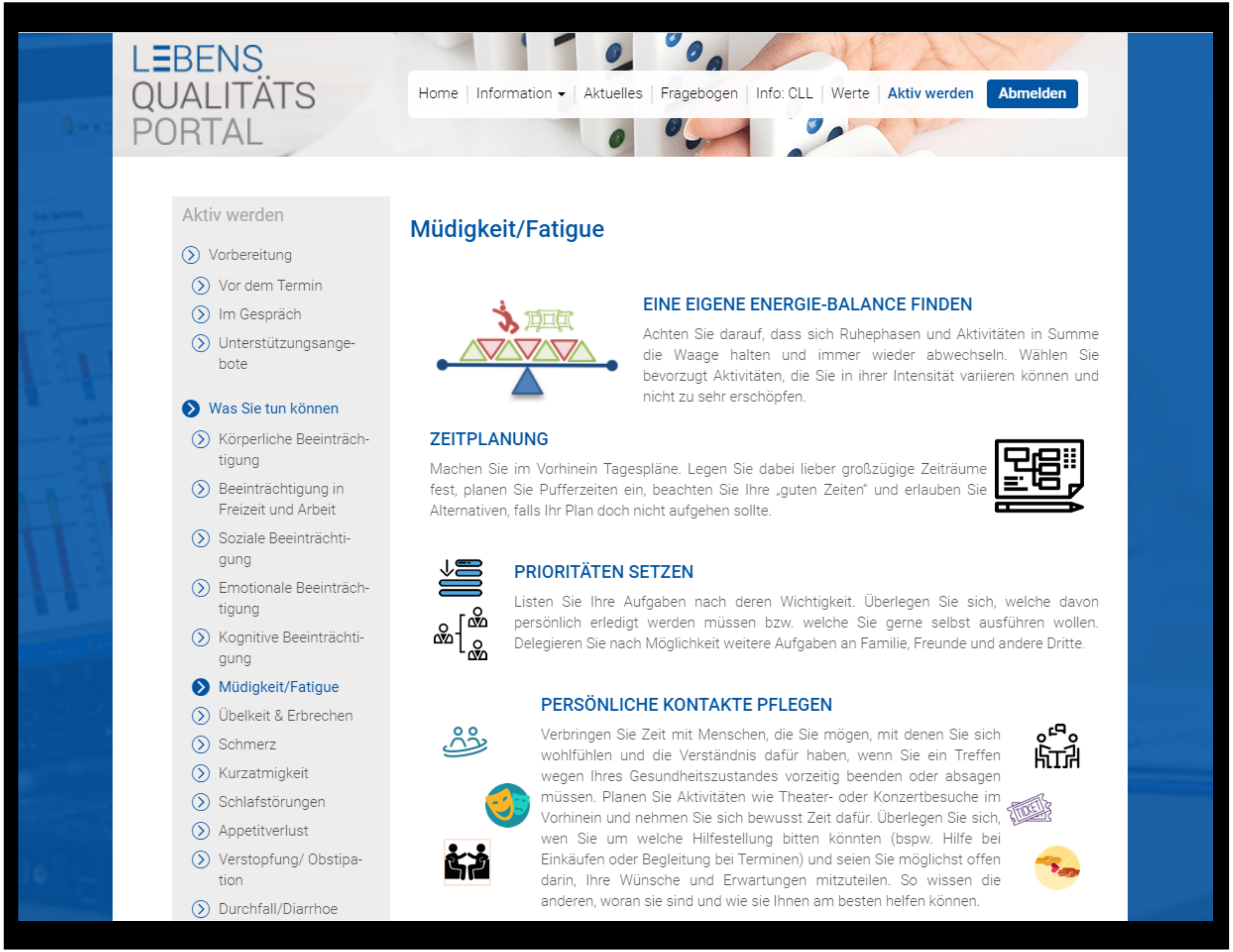

Aktiv werden

- Vorbereitung
  - Vor dem Termin
  - Im Gespräch
  - Unterstützungsangebote
- Was Sie tun können
  - Körperliche Beeinträchtigung
  - Beeinträchtigung in Freizeit und Arbeit
  - Soziale Beeinträchtigung
  - Emotionale Beeinträchtigung
  - Kognitive Beeinträchtigung
  - Müdigkeit/Fatigue
  - Übelkeit & Erbrechen
  - Schmerz
  - Kurzatmigkeit
  - Schlafstörungen
  - Appetitverlust
  - Verstopfung/ Obstipation
  - Durchfall/Diarrhoe

Müdigkeit/Fatigue

EINE EIGENE ENERGIE-BALANCE FINDEN

Achten Sie darauf, dass sich Ruhephasen und Aktivitäten in Summe die Waage halten und immer wieder abwechseln. Wählen Sie bevorzugt Aktivitäten, die Sie in ihrer Intensität variieren können und nicht zu sehr erschöpfen.

ZEITPLANUNG

Machen Sie im Vorhinein Tagespläne. Legen Sie dabei lieber großzügige Zeiträume fest, planen Sie Pufferzeiten ein, beachten Sie Ihre „guten Zeiten“ und erlauben Sie Alternativen, falls Ihr Plan doch nicht aufgehen sollte.

PRIORITÄTEN SETZEN

Listen Sie Ihre Aufgaben nach deren Wichtigkeit. Überlegen Sie sich, welche davon persönlich erledigt werden müssen bzw. welche Sie gerne selbst ausführen wollen. Delegieren Sie nach Möglichkeit weitere Aufgaben an Familie, Freunde und andere Dritte.

PERSÖNLICHE KONTAKTE PFLEGEN

Verbringen Sie Zeit mit Menschen, die Sie mögen, mit denen Sie sich wohlfühlen und die Verständnis dafür haben, wenn Sie ein Treffen wegen Ihres Gesundheitszustandes vorzeitig beenden oder absagen müssen. Planen Sie Aktivitäten wie Theater- oder Konzertbesuche im Vorhinein und nehmen Sie sich bewusst Zeit dafür. Überlegen Sie sich, wen Sie um welche Hilfestellung bitten könnten (bspw. Hilfe bei Einkäufen oder Begleitung bei Terminen) und seien Sie möglichst offen darin, Ihre Wünsche und Erwartungen mitzuteilen. So wissen die anderen, woran sie sind und wie sie Ihnen am besten helfen können.
