# Supplementary figures and images for: Using the Computer-based Health Evaluation System (CHES) to Support Self-management of Symptoms and Functional Health: Evaluation of Hematological Patient Use of a Web-Based Patient Portal
Source: J Med Internet Res. 2021 Jun 8;23(6):e26022. doi: 10.2196/26022 (PMC8262597; doi:10.2196/26022)

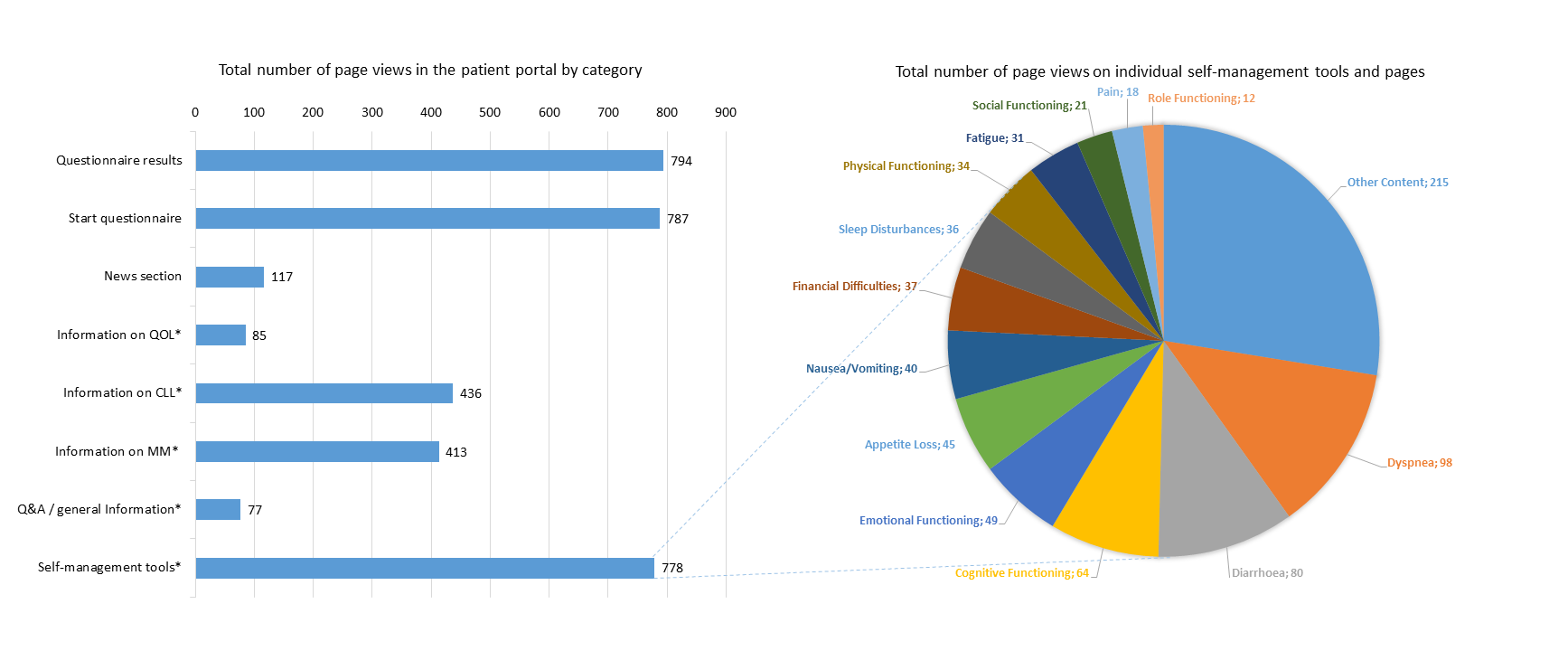

Supplement: Multimedia Appendix 2 [file jmir_v23i6e26022_app2.png]
